# Magnetic Resonance Imaging in the Year Prior to Total Knee Arthroplasty: A Potential Overutilization of Healthcare Resources

**DOI:** 10.5435/JAAOSGlobal-D-22-00262

**Published:** 2023-05-19

**Authors:** Katelyn E. Rudisill, Philip P. Ratnasamy, Peter Y. Joo, Lee E. Rubin, Jonathan N. Grauer

**Affiliations:** From the Department of Orthopedics and Rehabilitation, Yale School of Medicine, New Haven, CT.

## Abstract

**Background:**

Total knee arthroplasty (TKA) is a common procedure for late-stage degenerative changes, a situation for which magnetic resonance imaging (MRI) is typically not considered useful. In an era attempting to contain healthcare expenditures, the rate, timing, and predictors for MRI before TKA were assessed in a large, national, administrative data set.

**Methods:**

The 2010 to Q3 2020 MKnee PearlDiver data set was used to identify patients undergoing TKA for osteoarthritis. Those with lower extremity MRI for knee indications within 1 year before TKA were then defined. Patient age, sex, Elixhauser Comorbidity Index, region in the country, and insurance plan were characterized. Predictors of having had an MRI were assessed by univariate and multivariate analyses. The costs and timing of the obtained MRIs were also assessed.

**Results:**

Of 731,066 TKAs, MRI was obtained within 1 year prior for 56,180 (7.68%) with 28,963 (51.9%) within the 3 months of TKA. Independent predictors of having had an MRI included younger age (odds ratio [OR], 0.74 per decade increase), female sex (OR, 1.10), higher Elixhauser Comorbidity Index (OR, 1.15), region of the country (relative to South, Northeast OR, 1.08, West OR, 1.22, Midwest OR, 1.36), and insurance (relative to Medicare, Medicaid OR, 1.36 and Commercial OR, 1.35) with *P* < 0.0001 for each. The total cost of MRIs among patients who received a TKA is $44,686,308.

**Conclusion:**

Noting that TKA is typically done for advanced degenerative changes, MRI should rarely be indicated in the preoperative period for this procedure. Nonetheless, this study found that MRI was done within the year before TKA for 7.68% of the study cohort. In an era striving for evidence-based medicine, the almost $45 million dollars spent on MRI in the year before TKA may represent overutilization.

Total knee arthroplasty (TKA) is a common procedure for late-stage degenerative changes.^1^ This is a situation for which magnetic resonance imaging (MRI) is typically not considered useful, but its utilization in this patient population has not been well characterized.

Radiographs are the imaging modality of choice for assessing overall knee osteoarthritis and for tracking its progression.^2,3^ Such imaging, especially when weight-bearing radiographs are obtained, is a simple and cost-effective to determine joint space narrowing, osteophyte formation, subchondral sclerosis, cyst formation, and alignment.^2,3^ Radiological classification systems have been described and validated to delineate severity and disease progression, with the Kellgren and Lawrence classification being the most commonly used.^4^

MRI is an additional imaging modality that may be considered to gain information about menisci, ligaments, chondral surfaces, and early degenerative changes that are not well differentiated by radiographs.^2,5,6,7,8^ Although knee MRI is frequently used in younger patients for whom soft-tissue procedures may be considered,^7,9^ it is typically not needed for those with advanced degenerative changes of the knee for whom TKA is considered.^10^

In assessing healthcare utilization, several studies have suggested MRI overutilization, particularly in the setting of osteoarthritis.^8,11,12,13,14^ Specifically, Newman et al^8^ highlighted the limitations of MRIs to accurately represent conditions of the knee in patients diagnosed with osteoarthritis. Furthermore, Karel et al^11^ noted the minimal clinical benefit in patient outcomes provided by diagnostic imaging (MRI and CT) for patients with knee or low back pain. In addition, the Damask Trial Team found a negative cost benefit for the utilization of MRIs for musculoskeletal problems.^12^

During a time when healthcare costs are increasing at an alarming rate,^15,16,17,18,19,20^ there is the question of how frequently MRIs are obtained in the year before TKA, an area of potential overutilization. This study aims to assess the rate, timing, and predictors for knee MRI use before TKA in a large, national administrative data set.

## Methods

### Database and Cohort

This study used the 2010 through Q3 2020 MKnee PearlDiver data set (a national administrative multi-insurance database). The MKnee PearlDiver data set contains deidentified health information for more than two million orthopaedic patients, who falls within the guidelines outlined by the Health Insurance Portability and Accountability Act. As such, our Institutional Review Board granted studies using this data set exemption from a review.

TKA patients were identified based on Current Procedural Terminology code 27477. The cohort was then limited to those with a primary diagnosis of knee osteoarthritis using International Classification of Disease codes.

The occurrence of MRI within a year before TKA was then determined based on the CPT codes for lower extremity MRI without, with, and with/without contrast (73721, 73722, and 73723, respectively). As such codes are for “any joint of the lower extremity,” those of the knee were isolated by the primary diagnosis being any knee-related International Classification of Disease code.

### Patient Characteristics

Patient characteristics were then extracted. These included age, sex, Elixhauser Comorbidity Index (ECI), region of the country according to US Census Bureau definitions (West, South, Midwest, and Northeast), and insurance plan (Medicare, Medicaid, and Commercial).

The costs associated with MRIs performed were then assessed. Summary and total cost data were abstracted.

### Data Analysis

Descriptive analysis was used to characterize the timing of MRIs within the year before TKA. Patient characteristics of those without and with MRI within the year before MRI were then tabulated and compared. Univariate comparisons were done with the Pearson chi-squared test for categorical variables (sex, region, and insurance plan) and the Welch *t* test for continuous variables (age and ECI). Multivariate logistical regression was then done to assess independent predictors of having had an MRI in the year before TKA.

The PearlDiver system provided all statistical analyses. Statistical significance was set at *P* < 0.05 for all tests. Figures were created using Microsoft Excel (Microsoft) and Prism 9 (GraphPad Software).

## Results

### Timing of Magnetic Resonance Imaging Before Total Knee Arthroplasty

A total of 731,066 TKAs performed for osteoarthritis were identified for analysis. Of these, an MRI of the knee was obtained for 56,180 (7.68%) in the year before TKA.

**Figure 1:**
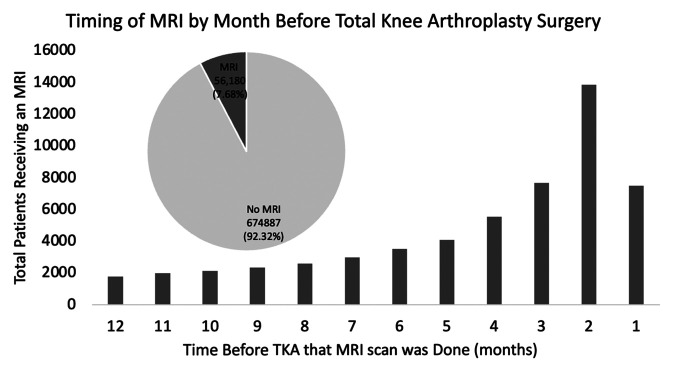
Diagram showing the timing of MRIs for patients receiving an MRI within 1 year before TKA is displayed for total patients receiving an MRI by the time before the TKA that the MRI was done in months. A pie graph is shown with the total patient population for those who received a TKA for this study cohort broken down by patients who did receive MRI within 1 year prior and those who did not receive an MRI within 1 year prior. MRI = magnetic resonance imaging, TKA = total knee arthroplasty

The timing by month before TKA was then characterized: 28,963 MRI scans were obtained within three months before the TKA, which constituted 51.9% of the MRIs obtained in the year before TKA (Figure 1). The incidence of MRIs gradually declined as one was further in advance of the TKA.

### Characterization of Those Who Did Not and Did Have Magnetic Resonance Imaging in the Year Prior Total Knee Arthroplasty

Characteristics of those who did and did not have MRI in the year before TKA are outlined in Table 1. By univariate analysis, those who had an MRI in the year before TKA were younger (average age 63.78 vs 66.42 years), more female (65.5% vs 62.8%), sicker (average ECI 5.00 vs 4.34), geographically different based on region of the country, and more likely to have Commercial or Medicare insurance (*P* < 0.0001 for each).

**Table 1:** Total Knee Arthroplasty Study Population and Univariate Comparison of Those With and Without MRI Within the Year Before Surgery

Demographic Factor	No MRI Within 1 yr Before TKA	MRI Within 1 yr Before TKA	*P* Value
N = 731,066	674,887 (92.32%)	56,180 (7.68%)	
Age (mean ± SD)	66.42 ± 8.62	63.76 ± 9.33	
<55	66,906 (9.9%)	9189 (16.4%)	**<0.0001**
55-64	206,112 (30.5%)	20,667 (36.8%)	
65-74	289,511 (42.9%)	18,642 (33.2%)	
>75	131,230 (19.4%)	8086 (14.4%)	
Sex			
Male	250,798 (37.2%)	19,403 (34.5%)	
Female	424,085 (62.8%)	36,777 (65.5%)	**<0.0001**
ECI (mean ± SD)	4.34 ± 3.20	5.00 ± 3.29	
0-1	125,290 (18.6%)	6706 (11.9%)	
2-3	189,687 (28.1%)	14,496 (25.8%)	**<0.0001**
4-5	157,474 (23.3%)	13,969 (24.9%)	
6-8	130,017 (19.3%)	12,935 (23%)	
>8	72,409 (10.7%)	8062 (14.4%)	
Region			
South	263,373 (39%)	18,882 (33.6%)	
Northeast	129,878 (19.2%)	10,415 (18.5%)	**<0.0001**
Midwest	195,005 (28.9%)	19,526 (34.8%)	
West	83,773 (12.4%)	6998 (12.5%)	
Insurance			
Medicare	228,692 (33.9%)	13,079 (23.3%)	
Medicaid	18,486 (2.7%)	2301 (4.1%)	**<0.0001**
Commercial	435,998 (64.6%)	41,121 (73.2%)	

ECI = Elixhauser Comorbidity Index, MRI = magnetic resonance imaging, SD = standard deviation, TKA = total knee arthroplasty. Bold p-values represent significance less than 0.05.

**Figure 2:**
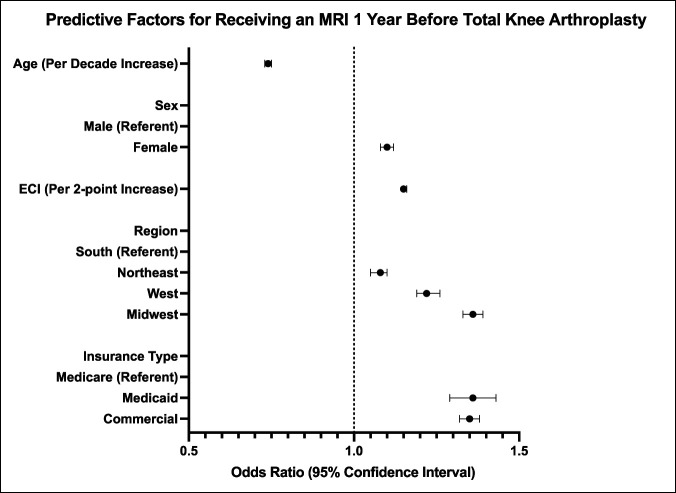
Forest plot of the predictive factors for patients who received an MRI within 1 year before surgery. Values for the plot are presented in Table 2. All points on the plot are statistically significant at a *P*-value of <0.05. MRI = magnetic resonance imaging

Multivariate analysis results are presented in Table 2 and Figure 2. MRI utilization within 1 year of TKA was independently associated with younger age (odds ratio [OR], 0.74 per decade increase), female sex (OR, 1.10), higher ECI (OR, 1.15), region of the country (relative to South, Northeast OR, 1.08, West OR, 1.22, and Midwest OR, 1.36), and insurance (relative to Medicare, Medicaid OR, 1.36 and Commercial OR, 1.35) (*P* < 0.0001 for each).

**Table 2:** Multivariate Analysis of Predictive Factors for Receiving an MRI 1 Year Before TKA

N = 731,066	OR (95% CI)	*P* Value
Age (per decade increase)	0.74 (0.73-0.75)	**<0.0001**
Sex		
Male (referent)		
Female	1.10 (1.08-1.12)	**<0.0001**
ECI (per two-point increase)	1.15 (1.15-1.16)	**<0.0001**
Region		
South (referent)		
Northeast	1.08 (1.05-1.1)	**<0.0001**
West	1.22 (1.19-1.26)	**<0.0001**
Midwest	1.36 (1.33-1.39)	**<0.0001**
Insurance		
Medicare (referent)		
Medicaid	1.36 (1.29-1.43)	**<0.0001**
Commercial	1.35 (1.32-1.38)	**<0.0001**

CI = confidence interval, ECI = Elixhauser Comorbidity Index, MRI = magnetic resonance imaging, OR = odds ratio, TKA = total knee arthroplasty

### Cost Associated With Magnetic Resonance Imagings

The average (inner quartile) cost of knee MRIs in the study was found to be $686.52 (range: $257.00 to $1057.00). Using this average cost, the total cost associated with the MRIs assessed in the study was $44,686,308.00.

## Discussion

TKA is one of the most common joint replacement procedures performed for late-stage degeneration.^21^ As such, MRI is generally not considered useful before this procedure.^22^ In fact, MRI before TKA may contribute to excess healthcare costs—a major issue in today's healthcare landscape.^23,24,25,26^

Of 731,066 TKA patients identified, 56,180 (7.68%) received an MRI within 1 year before TKA. Of the MRIs performed for this study cohort, 28,963 (51.9%) occurred in the 3 months immediately before the TKA, with rates declining further before TKA. These data show that a substantial number of patients with knee osteoarthritis received an MRI before TKA—with the timing of these MRIs suggesting that they were done for preoperative evaluation.

Multivariate analysis identified several independent predictors of receiving an MRI before TKA. In summary, younger, female, higher patients with ECI from certain geographies and with defined insurances were more likely to have had an MRI in the year before TKA.

Younger patients being more likely to have had an MRI in the year before TKA (OR, 0.74 per decade increase) seems to suggest that early degenerative findings may be thought to be better characterized in this population or that they are suspicious for soft-tissue issues.^27,28,29,30^ Female sex was another related variable (OR, 1.10). Interestingly, a back pain study similarly found a female predominance and questioned this to be a variable associated with having a lower threshold for obtaining such a study in this patient population.^24^ This correlation could also be related to female patients being more likely to sustain soft-tissue injuries of the knee where MRI imaging is indicated.^31-33^ ECI was yet another factor associated with obtaining an MRI before TKA (OR, 1.15 per two-point increase), but the reasons for this are unclear.

Geographic variations in MRI usage before TKA were noted, with patients in the Northeast, West, and Midwest being more likely to receive an MRI before surgery when compared with patients in the South (OR, 1.08, 1.22, and 1.36, respectively). This finding is in line with previous literature highlighting notable geographic variation in utilization of radiographic imaging for various pathologies and may be contributed to by local practice patterns.^34,35^ Finally, patients with Medicaid or commercial insurance were more likely to receive an MRI before TKA compared with patients with Medicare insurance (ORs, 1.36 and 1.35, respectively). Variations in reimbursement schedules and preauthorization policies between insurance providers may contribute to differential use of MRI before TKA.^24,36^

The cost of pre-TKA knee MRIs identified in this study is large, accounting for $44,686,308.00 in potentially excess healthcare expenditures over the 10-year study period. For perspective—with the average true cost of treat-and-release emergency department visits estimated to be $420—excess expenditures on MRI before TKA equate to the cost of more than 106,396 ED visits.

There are several important limitations to note regarding this study. As a retrospective, administrative database, the accuracy of the data is dependent on the coding, but the dichotomous occurrence (or not) of MRI in the study period was not expected to be prone to error. Furthermore, the perceived indications for the imaging studies were not directly available.

In summary, out of a large national cohort of TKA patients, 7.68% received an MRI of the affected region in the year before surgery. This was associated with substantial costs that bear consideration in an effort to optimize healthcare utilization.

## References

[1] Hussain SM Neilly DW Baliga S Patil S Meek R: Knee osteoarthritis: A review of management options. Scott Med J 2016;61:7-16. 27330013 10.1177/0036933015619588

[2] Oo WM Linklater JM Hunter DJ: Imaging in knee osteoarthritis. Curr Opin Rheumatol 2017;29:86-95. 27755179 10.1097/BOR.0000000000000350

[3] Teichtahl AJ Wluka AE Davies-Tuck ML Cicuttini FM: Imaging of knee osteoarthritis. Best Pract Res Clin Rheumatol 2008;22:1061-1074. 19041077 10.1016/j.berh.2008.09.004

[4] Kohn MD Sassoon AA Fernando ND: Classifications in brief: Kellgren-lawrence classification of osteoarthritis. Clin Orthop Relat Res 2016;474:1886-1893. 26872913 10.1007/s11999-016-4732-4 PMC4925407

[5] Mittal S Pradhan G Singh S Batra R: T1 and T2 mapping of articular cartilage and menisci in early osteoarthritis of the knee using 3-Tesla magnetic resonance imaging. Pol J Radiol 2019;84:e549-e564. 32082454 10.5114/pjr.2019.91375 PMC7016502

[6] Crema MD Roemer FW Marra MD : Articular cartilage in the knee: Current MR imaging techniques and applications in clinical practice and research. Radiographics 2011;31:37-61. 21257932 10.1148/rg.311105084 PMC6939857

[7] Milone MT Shenoy K Pham H Jazrawi LM Strauss EJ: MRI analysis of peripheral soft tissue composition, not body mass index, correlates with outcomes following anterior cruciate ligament reconstruction. Knee Surg Sports Traumatol Arthrosc 2018;26:3711-3716. 29725746 10.1007/s00167-018-4966-7

[8] Newman S Ahmed H Rehmatullah N: Radiographic vs. MRI vs. arthroscopic assessment and grading of knee osteoarthritis - are we using appropriate imaging? J Exp Orthop 2022;9:2. 34978625 10.1186/s40634-021-00442-y PMC8724325

[9] Newman JS Newberg AH: Basketball injuries. Radiol Clin North Am 2010;48:1095-1111. 21094400 10.1016/j.rcl.2010.07.007

[10] Menashe L Hirko K Losina E : The diagnostic performance of MRI in osteoarthritis: A systematic review and meta-analysis. Osteoarthritis Cartilage 2012;20:13-21. 22044841 10.1016/j.joca.2011.10.003 PMC3934362

[11] Karel YHJM Verkerk K Endenburg S Metselaar S Verhagen AP: Effect of routine diagnostic imaging for patients with musculoskeletal disorders: A meta-analysis. Eur J Intern Med 2015;26:585-595. 26186812 10.1016/j.ejim.2015.06.018

[12] DAMASK Trial Team: Cost-effectiveness of magnetic resonance imaging of the knee for patients presenting in primary care. Br J Gen Pract 2008;58.e10-e16. 19000394 10.3399/bjgp08X342660 PMC2576309

[13] Sajid IM Parkunan A Frost K: Unintended consequences: Quantifying the benefits, iatrogenic harms and downstream cascade costs of musculoskeletal MRI in UK primary care. BMJ Open Qual 2021;10:e001287. 10.1136/bmjoq-2020-001287 PMC8256731 34215659

[14] Oudenaarde Kv Swart NM Bloem JL : General practitioners referring adults to MR imaging for knee pain: A randomized controlled trial to assess cost-effectiveness. Radiology 2018;288:170-176. 29664339 10.1148/radiol.2018171383

[15] Rudmik L Drummond M: Health economic evaluation: Important principles and methodology. Laryngoscope 2013;123:1341-1347. 23483522 10.1002/lary.23943

[16] Arce HE: How to face the rising costs of healthcare? Medicina (B Aires) 2019;79:529-533. 31864221

[17] Wammes JJG van der Wees PJ Tanke MAC Westert GP Jeurissen PPT: Systematic review of high-cost patients' characteristics and healthcare utilisation. BMJ Open 2018;8:e023113. 10.1136/bmjopen-2018-023113 PMC6129088 30196269

[18] Soley-Bori M Ashworth M Bisquera A : Impact of multimorbidity on healthcare costs and utilisation: A systematic review of the UK literature. Br J Gen Pract 2021;71:e39-e46. 33257463 10.3399/bjgp20X713897 PMC7716874

[19] Puig-Junoy J Ruiz Zamora A: Socio-economic costs of osteoarthritis: A systematic review of cost-of-illness studies. Semin Arthritis Rheum 2015;44:531-541. 25511476 10.1016/j.semarthrit.2014.10.012

[20] Stahmeyer JT Hamp S Zeidler J Eberhard S: Healthcare expenditure and the impact of age: A detailed analysis for survivors and decedents. Bundesgesundheitsblatt Gesundheitsforschung Gesundheitsschutz 2021;64:1307-1314. 34258630 10.1007/s00103-021-03385-y

[21] Canovas F Dagneaux L: Quality of life after total knee arthroplasty. Orthop Traumatol Surg Res 2018;104:S41-S46. 29183821 10.1016/j.otsr.2017.04.017

[22] Munshi M Davidson M MacDonald PB Froese W Sutherland K: The efficacy of magnetic resonance imaging in acute knee injuries. Clin J Sport Med 2000;10:34-39. 10695848 10.1097/00042752-200001000-00007

[23] Shrank WH Rogstad TL Parekh N: Waste in the US health care system: Estimated costs and potential for savings. JAMA 2019;322:1501-1509. 31589283 10.1001/jama.2019.13978

[24] Jahanmehr N Bigdeli AS Salari H Mokarami H KhodaKarim S Damiri S: Analyzing inappropriate magnetic resonance imaging (MRI) prescriptions and resulting economic burden on patients suffering from back pain. Int J Health Plann Manage 2019;34:e1437-e1447. 31271228 10.1002/hpm.2806

[25] Willemé P Dumont M: Machines that go 'ping': Medical technology and health expenditures in OECD countries. Health Econ 2015;24:1027-1041. 25070599 10.1002/hec.3089

[26] Murthy VNR Okunade AA: Determinants of U.S. health expenditure: Evidence from autoregressive distributed lag (ARDL) approach to cointegration. Econ Model 2016;59:67-73.

[27] Neogi T Zhang Y: Epidemiology of osteoarthritis. Rheum Dis Clin North Am 2013;39:1-19. 23312408 10.1016/j.rdc.2012.10.004 PMC3545412

[28] Prieto-Alhambra D Judge A Javaid MK Cooper C Diez-Perez A Arden NK: Incidence and risk factors for clinically diagnosed knee, hip and hand osteoarthritis: Influences of age, gender and osteoarthritis affecting other joints. Ann Rheum Dis 2014;73:1659-1664. 23744977 10.1136/annrheumdis-2013-203355 PMC3875433

[29] Hewett TE Myer GD Ford KR Paterno MV Quatman CE: Mechanisms, prediction, and prevention of ACL injuries: Cut risk with three sharpened and validated tools. J Orthop Res 2016;34:1843-1855. 27612195 10.1002/jor.23414 PMC5505503

[30] LaBella CR Hennrikus W Hewett TE : Anterior cruciate ligament injuries: Diagnosis, treatment, and prevention. Pediatrics 2014;133:e1437-e1450. 24777218 10.1542/peds.2014-0623

[31] Boden BP Sheehan FT Torg JS Hewett TE: Noncontact anterior cruciate ligament injuries: Mechanisms and risk factors. Am Acad Orthop Surg 2010;18:520-527. 10.5435/00124635-201009000-00003 PMC3625971 20810933

[32] Acevedo RJ Rivera-Vega A Miranda G Micheo W: Anterior cruciate ligament injury: Identification of risk factors and prevention strategies. Curr Sports Med Rep 2014;13:186-191. 24819011 10.1249/JSR.0000000000000053

[33] Sutton KM Bullock JM: Anterior cruciate ligament rupture: Differences between males and females. J Am Acad Orthop Surg 2013;21:41-50. 23281470 10.5435/JAAOS-21-01-41

[34] Bhargavan M Sunshine JH: Utilization of radiology services in the United States: Levels and trends in modalities, regions, and populations. Radiology 2005;234:824-832. 15681686 10.1148/radiol.2343031536

[35] Shraim M Cifuentes M Willetts JL Marucci-Wellman HR Pransky G: Why does the adverse effect of inappropriate MRI for LBP vary by geographic location? An exploratory analysis. BMC Musculoskelet Disord 2019;20:574. 31785613 10.1186/s12891-019-2964-7 PMC6885323

[36] Smith HE Mistovich RJ Cruz AI Jr : Does insurance status affect treatment of children with tibial spine fractures? Am J Sports Med 2021;49:3842-3849. 34652247 10.1177/03635465211046928

